# Progranulin deficiency leads to severe inflammation, lung injury and cell death in a mouse model of endotoxic shock

**DOI:** 10.1111/jcmm.12756

**Published:** 2016-01-12

**Authors:** Yuan Yu, Xiaoying Xu, Lu Liu, Sheng Mao, Tingting Feng, Yi Lu, Yizhe Cheng, Hongyan Wang, Weiming Zhao, Wei Tang

**Affiliations:** ^1^ Department of HematologyQilu HospitalShandong UniversityJinanShandongChina; ^2^ Department of Pathogenic BiologyShandong University School of MedicineJinanShandongChina; ^3^ Department of PathologyHuai'an First People's HospitalHuai'anJiangsuChina; ^4^ Department of Biochemistry and Molecular BiologyShandong University School of MedicineJinanShandongChina; ^5^ Department of Medical MicrobiologyWeifang Medical UniversityWeifangShandongChina

**Keywords:** progranulin, lipopolysaccharide, endotoxic shock, inflammation

## Abstract

Progranulin (PGRN) is a crucial secreted growth factor involved in various kinds of physiologic and disease processes and often has a protective role in inflammatory diseases. This study was designed to investigate the protective effects of PGRN on endotoxic shock in a mouse model of PGRN deficiency. After lipopolysaccharide (LPS) injection to induce endotoxic shock in mice, PGRN levels were induced in wild‐type (WT) mice at 6 and 24 hrs. Survival rate analysis, haematoxylin and eosin staining, immunohistochemical staining, enzyme‐linked immunosorbent assay and *in situ* terminal deoxynucleotidyl transferase–mediated uridine triphosphate nick‐end labelling assay were used to reveal the susceptibility, lung injury, inflammatory cell infiltration, production of inflammatory mediators and lung cell death in mice after LPS injection. PGRN‐deficient (*Grn* ^−/−^) mice were highly susceptible to LPS‐induced endotoxic shock, with decreased survival, severe lung injury, increased production of pro‐inflammatory mediators, and inflammatory cell infiltration and apoptotic death in the lung. Additionally, recombinant PGRN (rPGRN) administration before LPS stimulation ameliorated the survival of and abnormalities in both WT and *Grn* ^−/−^ mice. Altogether, these findings indicate that PGRN may be a novel biologic agent with therapeutic potential for endotoxic shock probably by inhibiting LPS‐induced systemic and local inflammation in mice for treating endotoxic shock.

## Introduction

Endotoxic shock, a type of acute inflammatory reaction, is the second most common cause of non‐cardiac mortality. Patients typically present systemic hypotension, hyporeactiveness to vasoconstrictors and organ dysfunction 1. The pathogenesis of endotoxic shock is highly complicated; the most widely recognized cause is exposure to lipopolysaccharide (LPS), a major constituent of the cell wall of Gram‐negative bacteria 2. Toll‐like receptor 4 (TLR4) is the principal receptor in the inflammatory response to LPS 3. Toll‐like receptor 4 signal transduction stimulated by LPS initiates a complex signalling cascade of factors against pathogens, including mitogen‐activated protein kinases, nuclear factor‐kappa B (NF‐κB) and activator protein 1 (AP‐1) and the production of downstream pro‐inflammatory cytokines, chemokines or leucocyte adhesion molecules 4. The release of these inflammatory mediators triggers multi‐organ failure, leucocyte infiltration, increased blood flow and the generation of oxygen‐free radicals 5, 6. Their inhibition protected against Gram‐negative endotoxic shock in an experimental model 7.

Progranulin (PGRN), also known as granulin–epithelin precursor, contains 7^1/2^ repeats of a cysteine‐rich motif and forms a unique ‘beads‐on‐a‐string’ structure 8. Progranulin is abundantly expressed in epithelial cells, cells of the immune system and neurons and is likewise expressed in a broad range of other tissues and cell types, such as skeletal muscle, chondrocytes, adipose tissue and hematopoietic cells 9, 10. Progranulin plays a critical role in various physiological processes, including early embryogenesis, wound healing and host‐defence responses. In addition, PGRN is widely involved in the pathogenesis of many types of diseases, such as autoimmune disorders, cancer, atherosclerosis, obesity and neurodegenerative diseases 11, 12, 13, 14, 15, 16.

Recent studies have highlighted the importance of PGRN in acute and chronic inflammation. In macrophages from PGRN‐deficient mice, interleukin 10 (IL‐10) level was lower and inflammatory cytokine levels were higher than those in wild‐type (WT) mice on exposure to LPS, and recombinant PGRN (rPGRN) inhibited LPS‐mediated cytokine release from macrophages 17. In PGRN‐deficient (*Grn* ^−/−^) mice, *Listeria monocytogenes* infection was cleared less quickly than in WT mice 17. We recently reported that in a mouse model of renal ischaemia/reperfusion injury, *Grn* ^−/−^ mice showed significantly aggravated renal injury, and administration of rPGRN protected against renal ischaemia/reperfusion injury 18.

Although the anti‐inflammatory role of PGRN has been extensively recognized, systemic inflammation and tissue damage induced by endotoxin under PGRN deficiency are still not well elucidated. Here, we aimed to investigate the role of PGRN in defending against endotoxic shock in *Grn* ^−/−^ mouse. As compared with WT mice, PGRN‐deficient mice were extremely susceptible to LPS loading because of exaggerated inflammatory responses, and PGRN supplementation in WT and *Grn* ^−/−^ mice resulted in resistance to endotoxic shock. Our studies support that PGRN is a key protector in endotoxic shock, among other inflammatory diseases.

## Materials and methods

### Mice

Progranulin‐deficient mice (B6(Cg)‐*Grn* ^*tm1.1Aidi*^/J) were purchased from the Jackson Laboratory (Bar Harbor, ME, USA) and were backcrossed with a C57BL/6 background for at least six generations before use in the experiments. The WT mice in the present study were C57BL/6 obtained from Vital River Laboratories (Beijing, China). The experimental protocols were approved by the Institutional Animal Care and Use Committee of Shandong University (Permit Number: KYLL‐2013‐112). The investigation conforms to the US National Institutes of Health Guide for the Care and Use of Laboratory Animals and was performed in accordance with the ARRIVE guidelines (http://www.nc3rs.org/ARRIVE). All mice were housed under specific pathogen‐free conditions and maintained on a 12‐hr light/dark cycle at 25 ± 2°C, with free access to food and water. All surgery was performed under sodium pentobarbital anaesthesia (50 mg/kg, i.p.), and every effort was made to minimize suffering.

### Murine model of endotoxic shock

WT or *Grn* ^−/−^ mice (male, 8 weeks of age, weight 20–24 g) were intraperitoneally injected with LPS (*Escherichia coli* 055:B5; Sigma‐Aldrich, St. Louis, MO, USA) at 0, 2.5, 5, 10, 25, 35 or 50 mg/kg. Mice were monitored for survival for up to 5 days at an 8‐hr interval. In parallel experiments, 6 and 24 hrs after LPS injection, blood was obtained *via* cardiac puncture from WT and *Grn* ^−/−^ mice with LPS at 25 mg/kg under sodium pentobarbital (50 mg/kg, i.p.) anaesthesia, then mice were killed by cervical dislocation and the superior lobe of the right lung was excised from mice.

To further implicate the protective role of PGRN in endotoxic shock, WT or *Grn* ^−/−^ mice were pre‐treated with human rPGRN (generated and purified as reported 18, 19) at 10 mg/kg by intraperitoneal injection 2 hrs before intraperitoneal injection with LPS at 35 mg/kg. WT or *Grn* ^−/−^ mice of vehicle groups were administered PBS by intraperitoneal injection 2 hrs before LPS injection. Mice were monitored for survival for up to 5 days at a 8‐hr interval. Blood and lung tissues were obtained from mice at 6 and 16 hrs after LPS injection.

### Cell culture and treatments

Adenocarcinomic human alveolar basal epithelial A549 cells (American Type Culture Collection, Manassas, VA, USA) were cultured in DMEM (Invitrogen, Carlsbad, CA, USA) containing 10% FBS (Invitrogen), 100 IU/ml penicillin and 100 μg/ml streptomycin (Sigma‐Aldrich). A549 cells were pre‐treated with 500 ng/ml rPGRN or PBS 1 hr before 100 ng/ml LPS was added, and total RNA was isolated at 0, 2, 4, 8 and 12 hrs after LPS treatment. Bone marrow derived macrophages (BMDMs) were isolated from WT and *Grn* ^−/−^ mice as described previously 12. Wild‐type and *Grn* ^−/−^ BMDMs were pre‐treated with 500 ng/ml rPGRN or PBS 1 hr before 100 ng/ml LPS was added. At 24 hrs after LPS treatment, culture medium grown with BMDMs was collected.

### RNA extraction and real‐time RT‐PCR

Total RNA was isolated from mouse lungs or A549 cells by use of TRIzol reagent according to the manufacturer's instructions (Life Technologies, Carlsbad, CA, USA). Then, 1 μg of DNA‐free total RNA was reverse transcribed by use of a one‐step RT‐PCR kit (TaKaRa Bio, Shiga, Japan). The following sequence‐specific primers were synthesized: 5′‐GGTTGATGGTTCGTGGGGATGTTG‐3′ and 5′‐AAGGCAAAGACACTGCCCTGTTGG‐3′ for mouse PGRN; 5′‐GAAAAGCAAGCAGCCAACCA‐3′ and 5′‐CGGATCATGCTTTCTGTGCTC‐3′ for mouse tumour necrosis factor α (TNF‐α); 5′‐CTGCAGCTGGAGAGTGTGG‐3′ and 5′‐GGGGAACTCTGCAGACTCAA‐3′ for mouse IL‐1β; 5′‐AGTTGCCTTCTTGGGACTGA‐3′ and 5′‐TCCACGATTTCCCAGAGAAC‐3′ for mouse IL‐6; 5′‐GGTTGCCAAGCCTTATCGGA‐3′ and 5′‐ACCTGCTCCACTGCCTTGCT‐3′ for mouse IL‐10; 5′‐GGCTGTATTCCCCTCCATCG‐3′ and 5′‐CCAGTTGGTAACAATGCCATGT‐3′ for mouse β‐actin; 5′‐CCTCTCTCTAATCAGCCCTCTG‐3′ and 5′‐GAGGACCTGGGAGTAGATGAG‐3′ for human TNF‐α; 5′‐ATGATGGCTTATTACAGTGGCAA‐3′ and 5′‐GTCGGAGATTCGTAGCTGGA‐3′ for human IL‐1β; 5′‐ACTCACCTCTTCAGAACGAATTG‐3′ and 5′‐CCATCTTTGGAAGGTTCAGGTTG‐3′ for human IL‐6; 5′‐CAGCCAGATGCAATCAATGCC‐3′ and 5′‐TGGAATCCTGAACCCACTTCT‐3′ for human monocyte chemoattractant protein 1 (MCP‐1); and 5′‐GAAGTGTGACGTGGACATCC‐3′ and 5′‐CCGATCCACACGGAGTACTT‐3′ for human β‐actin. Reactions were performed in a 50‐μl SYBR GREEN PCR volume in a 96‐well optical reaction plate formatted in the Bio‐Rad iCycler system (Bio‐Rad, Hercules, CA, USA). β‐actin was used as an internal control for RNA quality and differences among samples.

### Western blot assay

Protein samples prepared from lung tissues or serum of mice were quantified by the Bradford assay and then underwent 10% SDS‐PAGE and were electrotransferred to polyvinylidene difluoride membranes for 2 hrs at 100 V with a standard transfer solution. After being blocked with 10% non‐fat milk, membranes were incubated with primary antibody for PGRN (1:1000; Santa Cruz Biotechnology, Dallas, TX, USA), high mobility group box protein 1 (HMGB1; 1:1000; Abcam, Cambridge, MA, USA), Bax (1:1000; ProteinTech Group, Chicago, IL, USA), Bcl‐2 (1:1000; ProteinTech Group), caspase 3 (1:1000; ProteinTech Group), cytochrome c (1:1000; ProteinTech Group), poly (ADP‐ribose) polymerase (PARP; 1:1000; ProteinTech Group) and cold‐inducible RNA‐binding protein (CIRP; 1:200; ProteinTech Group), with β‐actin antibody as a control (1:6000; ProteinTech Group). Proteins were visualized by chemiluminescence with an ECL kit (Millipore Corp., Billerica, MA, USA).

### Immunohistochemical staining

Immunoreactivity of PGRN, neutrophils and macrophages was assessed by immunohistochemistry by staining of paraffin‐embedded lung tissue sections as described 20 with control IgG (1:100; Santa Cruz Biotechnology), PGRN antibody (1:100; Santa Cruz Biotechnology) and Ly‐6B.2 and CD68 antibodies (1:100; AbD Serotec, Oxford, UK).

### Histology

Formalin‐fixed lung sections were stained with haematoxylin and eosin for histology. Lung injury was evaluated as congestion, oedema and infiltration of inflammatory cells to grade the degree of lung injury in 4 high‐power fields (HPFs, ×200 magnification) per section for each sample. Each variable was scored in a blinded manner as 0, normal; 1, ≤25% injury; 2, 25–50%; 3, 50–75%; or 4, ≥75% 21.

### Detection of cytokines

Serum levels of TNF‐α, IL‐6, IL‐10 and MCP‐1 were measured by use of ELISA kits (mouse TNF‐α, IL‐6 and IL‐10 ELISA kits were from eBioscience, San Diego, CA, USA; mouse MCP‐1 ELISA kit was from DAKEWE, Beijing, China). The level of PGRN in serum and lung homogenates was measured by an ELISA kit (AdipoGen, San Diego, CA, USA).

### *In situ* terminal deoxynucleotidyl transferase–mediated uridine triphosphate nick‐end labelling assay

Lung cell apoptosis after LPS injection was detected by transferase–mediated uridine triphosphate nick‐end labelling (TUNEL) assay, which was performed according to the manufacturer's protocol (Roche Diagnostics, Mannheim, Germany).

### Statistical analysis

Data are represented as mean ± S.D. Differences were estimated by one‐way anova followed by Duncan's multiple range tests. Survival of mice after LPS injection was analysed by Kaplan–Meier survival analysis with the log‐rank test for between‐group comparisons. *P* < 0.05 was considered statistically significant.

## Results

### PGRN was increased in serum and lung in a mouse model of endotoxic shock

**Figure 1:**
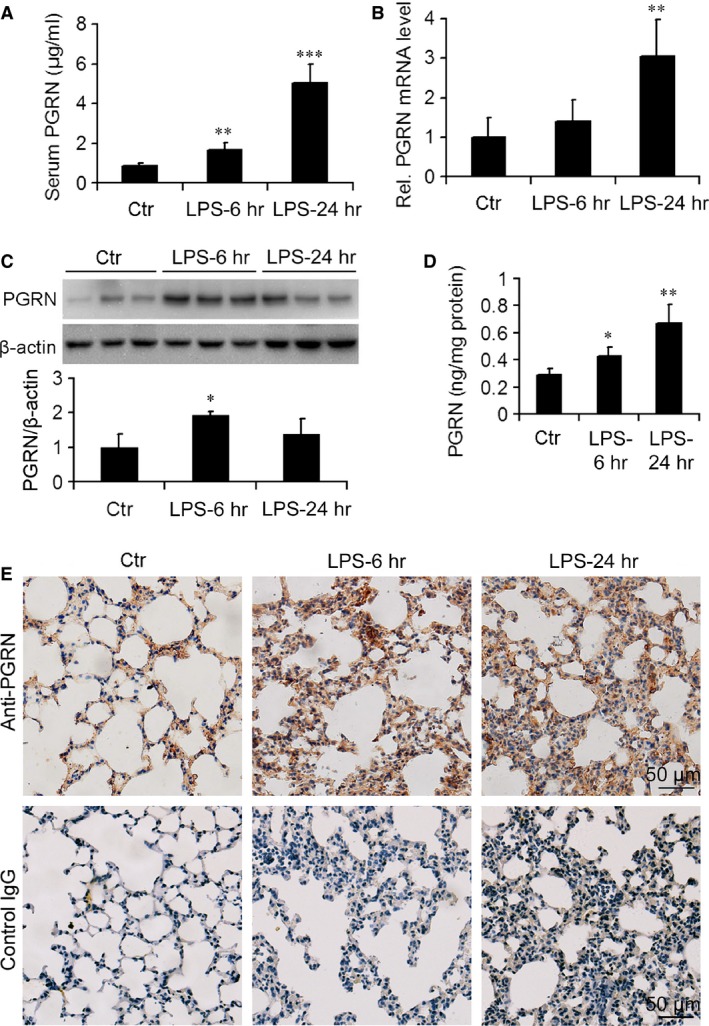
Level of progranulin (PGRN) was increased in wild‐type (WT) mice after lipopolysaccharide (LPS) injection. (**A**) Serum levels of progranulin (PGRN). (**B**) Real‐time RT‐PCR analysis of relative PGRN mRNA level in mouse lung. (**C**) Representative western blot gel documents and summarized data showing the PGRN protein level in mouse lung. (**D**) ELISA of level of PGRN in homogenates in mouse lung. (**E**) Representative photomicrographs of immunohistochemical staining of mouse lung tissue using antibody to PGRN or negative control IgG. Data are mean ± S.D. *P < 0.05; **P < 0.01; ***P < 0.001 compared with control WT mice without LPS injection (Ctr) (*n* = 6 mice/group).

We first detected the levels of PGRN in mice after LPS injection at 25 mg/kg. The serum level of PGRN in WT mice was higher with than without LPS injection (Fig. 1A). mRNA and protein levels of PGRN were enhanced in the lung after LPS administration at 6 and 24 hrs (Fig. 1B–D), which was further confirmed by immunohistochemical staining of lung tissue (Fig. 1E). Meanwhile, no staining with negative control IgG in the lung from mice was observed, indicating the specificity of the PGRN immunostaining.

### PGRN‐deficient mice were more susceptible to LPS than WT mice

**Figure 2:**
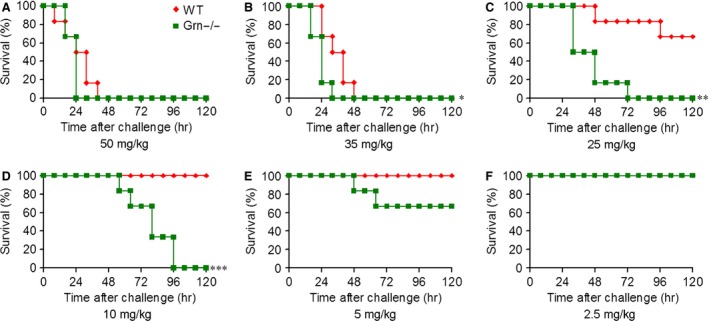
PGRN deficiency decreased the survival of mice after LPS injection. WT and *Grn* ^−/−^ mice were subjected to endotoxic shock induced by injection of LPS at 50 (**A**), 35 (**B**), 25 (**C**), 10 (**D**), 5 (**E**), or 2.5 mg/kg (**E**); survival was monitored 3 times/day for 5 days. *P < 0.05; **P < 0.01; ***P < 0.001 compared with WT mice injected with LPS (*n* = 6 mice/group).

To investigate the sensitivity of PGRN‐deficient mice to LPS‐induced shock, we administered LPS doses to WT and *Grn* ^−/−^ mice and noted survival rates. Lipopolysaccharide at 50 and 35 mg/kg resulted in 100% mortality of WT and *Grn* ^−/−^ mice within 48 hrs (Fig. 2). Progranulin deficiency accelerated endotoxic shock‐induced death with LPS at 35, 25 and 10 mg/kg (*P* < 0.05). Lipopolysaccharide at 10 and 5 mg/kg did not lead to the death of WT mice but resulted in 100% and 33.33% mortality of *Grn* ^−/−^ mice, respectively. In addition, WT and *Grn* ^−/−^ mice with LPS at 2.5 mg/kg showed no mortality. Thus, *Grn* ^−/−^ mice were exceedingly susceptible to endotoxic shock.

### PGRN deficiency exacerbated lung injury after LPS administration

**Figure 3:**
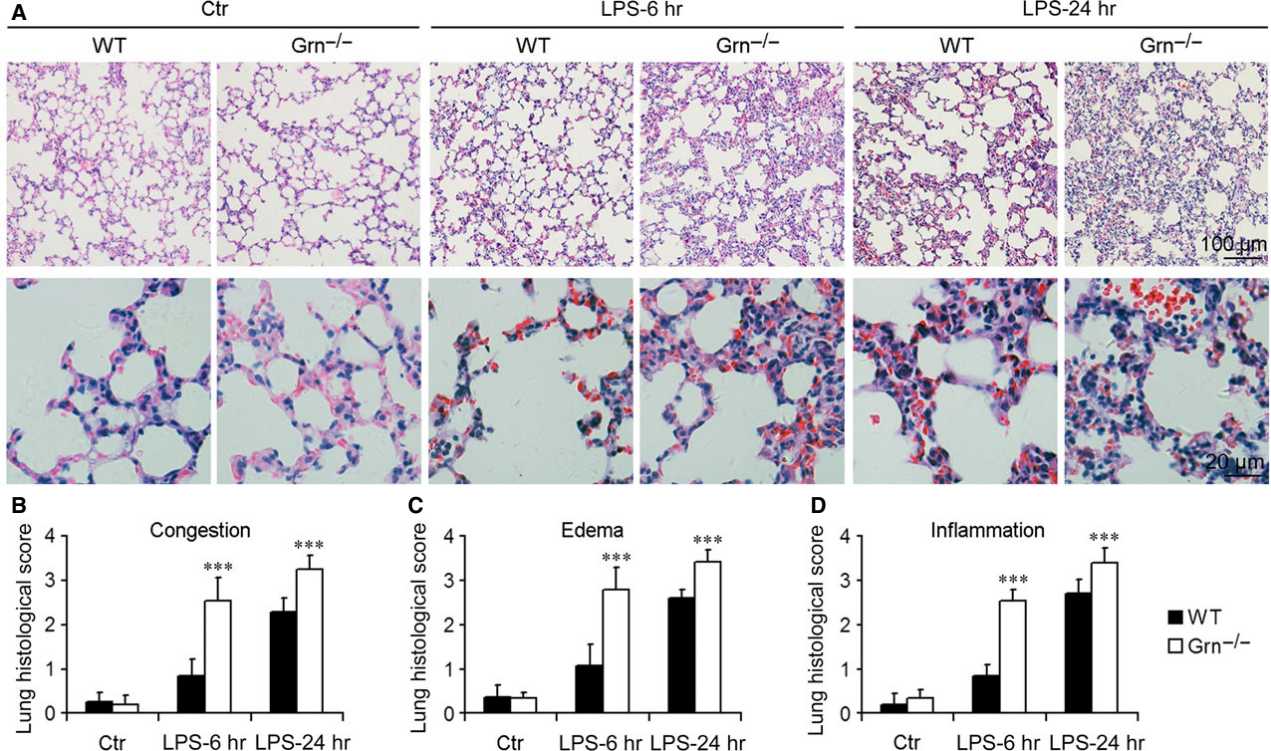
PGRN deficiency exacerbated lung injury after LPS injection at 25 mg/kg. (**A**) Representative micrographs of morphology of lungs from different groups of mice. Quantitative assessment of lung injury evaluated by congestion (**B**), oedema (**C**) and infiltration of inflammatory cells (**D**). Data are mean ± S.D. ***P < 0.001 compared with WT mice (*n* = 6 mice/group).

Haematoxylin and eosin–stained lung tissue sections from *Grn* ^−/−^ and WT mice with or without LPS at 25 mg/kg were compared to determine whether PGRN deficiency affected endotoxin‐induced lung injury. *Grn* ^−/−^ mice were phenotypically normal and had no appreciable defects in lung morphology. However, PGRN deficiency significantly aggravated lung injury in mice with LPS administration (Fig. 3A). Compared with WT mice, *Grn* ^−/−^ mice after LPS administration showed more severe morphological injury, as evidenced by enhanced congestion, interstitial oedema and inflammatory cell infiltration (Fig. 3B–D).

### Neutrophils and macrophages were accumulated in lungs of PGRN‐deficient mice after LPS administration

**Figure 4:**
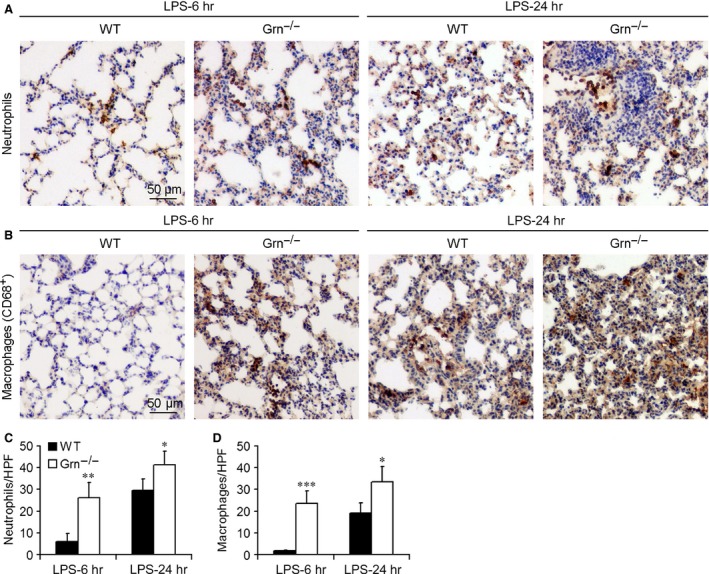
PGRN deficiency increased inflammatory cell infiltration in the mouse lung after injection of LPS. Representative sections of mouse lung stained for (**A**) neutrophils and (**B**) macrophages. Analysis of (**C**) neutrophil infiltrates (number/high‐power field [HPF]) and (**D**) macrophage infiltrates in the lung (number/HPF). Data are mean ± S.D. *P < 0.05; **P < 0.01; ***P < 0.001 compared with WT mice (*n* = 6 mice/group).

Neutrophils and macrophages are leucocytes that belong to innate immune system and are considered the main initial defenders against pathogens. We analysed the cell infiltrations in endotoxic lungs. Figure 4A and B shows representative immunohistochemistry staining of neutrophils and macrophages in lungs from WT and *Grn* ^−/−^ mice treated with LPS at 25 mg/kg. At 6 and 24 hrs after LPS treatment, neutrophil infiltration was greater in *Grn* ^−/−^ than WT lungs (Fig. 4C). Similarly, WT lungs showed few interstitial macrophages at 6 hrs after LPS administration but progressive accumulation at 24 hrs, whereas *Grn* ^−/−^ lungs showed pronounced macrophage infiltration at 6 and 24 hrs after LPS administration (Fig. 4D).

### Inflammatory cytokine levels were elevated in PGRN‐deficient mice after LPS administration

**Figure 5:**
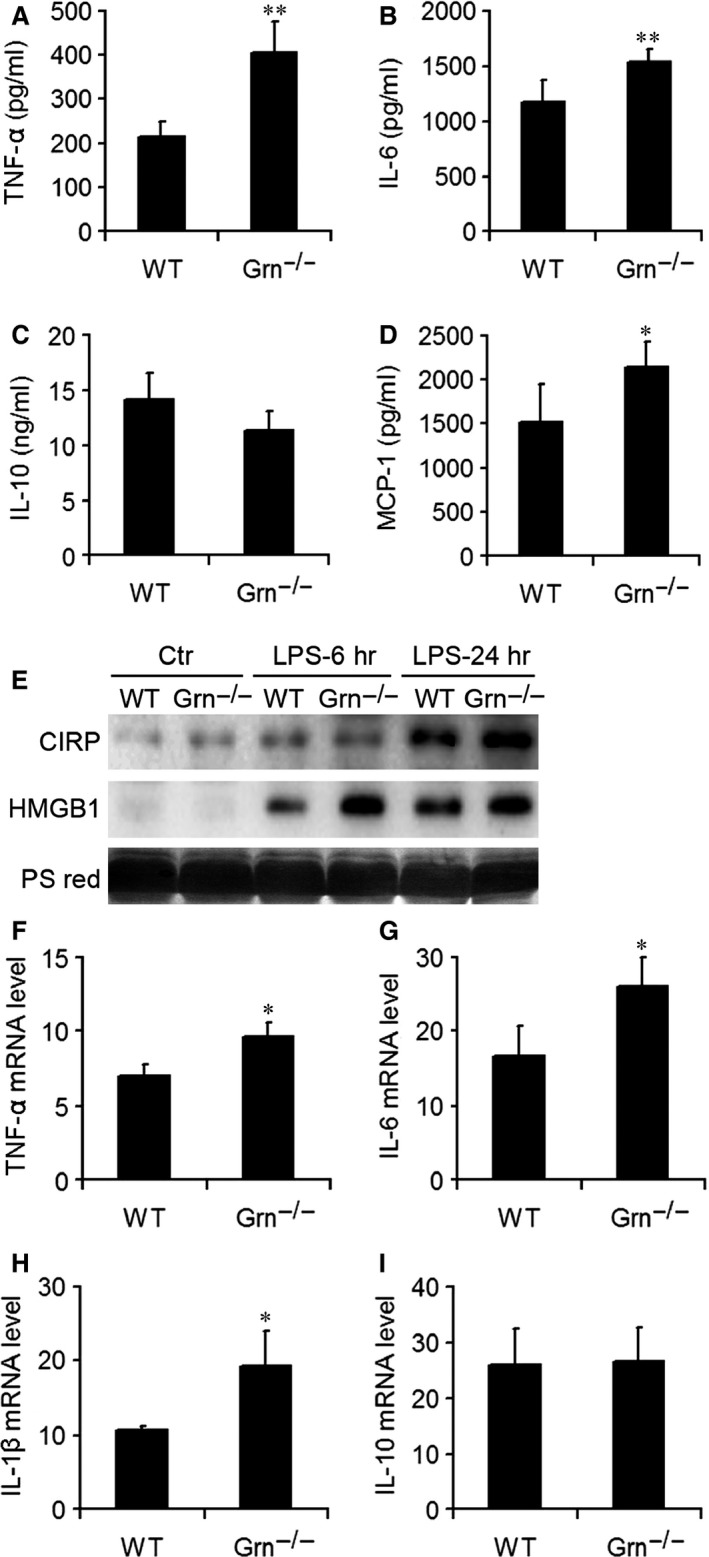
PGRN deficiency increased the production of systemic and local pro‐inflammatory mediators after injection of LPS. ELISA of serum levels of inflammatory mediators including tumour necrosis factor α (TNF‐α) (**A**), interleukin 6 (IL‐6) (**B**), IL‐10 (**C**) and monocyte chemoattractant protein 1 (MCP‐1) (**D**). (**E**) Western blot analysis of cold‐inducible RNA‐binding protein (CIRP) and high mobility group box protein 1 (HMGB1) in the serum from WT and *Grn* ^−/−^ mice with LPS injection at 6 and 24 hrs. PS red, Ponceau S red staining. Real‐time RT‐PCR analysis of mRNA levels of TNF‐α (**F**), IL‐6 (**G**), IL‐1β (**H**) and IL‐10 (**I**) in the lung after LPS injection. Data are mean ± S.D. *P < 0.05; **P < 0.01 compared with WT mice (*n* = 6 mice/group).

The pathophysiology of endotoxic shock is characterized by activation of multiple inflammatory genes and their products, which initiates the inflammatory process, organ dysfunction and mortality. We measured concentrations of inflammatory mediators in serum of mice to provide insights into the negative regulatory function of PGRN deficiency in endotoxic shock. As compared with WT mice, *Grn* ^−/−^ mice showed increased serum levels of TNF‐α, IL‐6 and MCP‐1 at 6 hrs after LPS injection at 25 mg/kg, with the level of anti‐inflammatory IL‐10 slightly decreased (Fig. 5A–D). Serum levels of CIRP and HMGB1 were determined by western blot. As shown in Figure 5E, the serum levels of CIRP and HMGB1 were increased at 6 and 24 hrs after LPS injection, and PGRN deficiency resulted in higher serum levels of CIRP and HMGB1. At 6 hrs after LPS injection, mRNA levels of TNF‐α, IL‐6, IL‐1β and IL‐10 were stimulated in WT lungs, and the levels of TNF‐α, IL‐6 and IL‐1β were higher in *Grn* ^−/−^ than in WT lungs (Fig. 5F–I). Elevated inflammatory cell infiltration and production of inflammatory mediators indicated that loss of PGRN signalling exacerbated systemic and local inflammation respond to LPS treatment.

### Apoptosis of lung cells was enhanced in PGRN‐deficient mice after LPS administration

**Figure 6:**
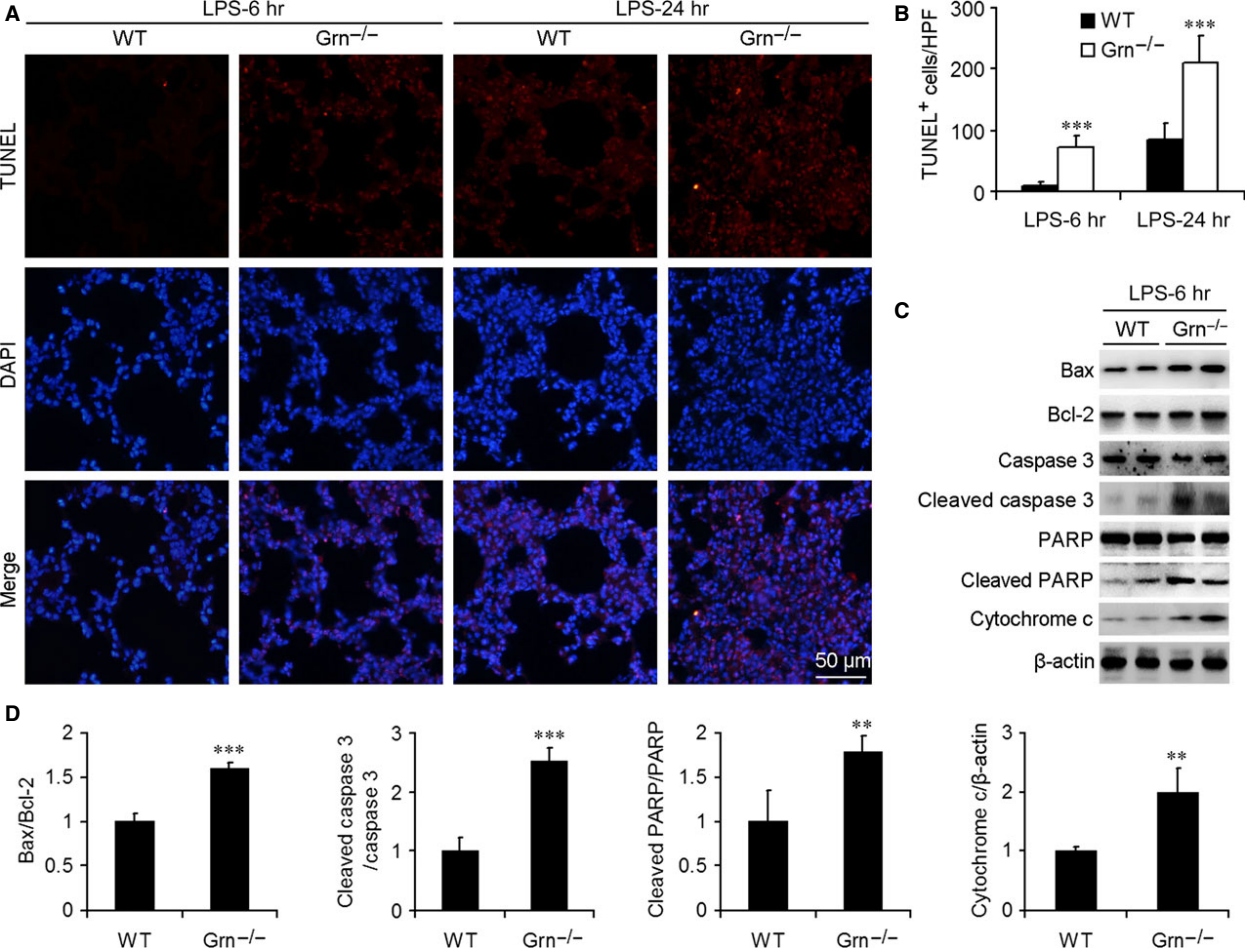
Effect of PGRN deficiency on lung cell apoptosis and apoptosis‐associated protein expression in endotoxic mouse model. (**A**) TUNEL assay was performed to assess lung cell death. Nuclei were revealed by 4′,6‐diamidino‐2‐phenylindole (DAPI) staining. (**B**) Quantification of number of TUNEL‐positive (dead) cells (number/HPF). Data are mean ± S.D. ***P < 0.001 compared with WT mice injected with LPS at 25 mg/kg (*n* = 6 mice/group). (**C**) Western blot analysis of protein levels of Bax, Bcl‐2, caspase 3, cytochrome c and poly (ADP‐ribose) polymerase (PARP) with LPS injection at 6 hrs. β‐actin was a loading control. (**D**) Summarized data of Bax/Bcl‐2, cleaved caspase 3/caspase 3, cleaved PARP/PARP and cytochrome c/β‐actin ratio in lung from WT and *Grn* ^−/−^ mice with LPS injection at 6 hrs. Data are mean ± S.D. **P < 0.01; ***P < 0.001 compared with WT mice.

To examine whether PGRN deficiency affected the tissue cell viability after LPS injection, we assessed lung apoptosis by TUNEL assay. TUNEL‐positive cells were observed in lungs of WT mice at 24 hrs after LPS administration but were substantially increased in number in lungs of PGRN‐deficient mice at 6 hrs after LPS administration (Fig. 6A). Moreover, TUNEL‐positive cells were markedly higher in *Grn* ^−/−^ than in WT lungs at 6 and 24 hrs after LPS injection (Fig. 6B). We further detected the levels of important proteins involved in apoptotic pathways. The protein levels of Bax, cleaved caspase 3, cytochrome c and cleaved PARP1 were greater in *Grn* ^−/−^ than in WT lungs at 6 hrs after LPS administration (Fig. 6C and D), which suggested that PGRN deficiency led to increased apoptosis in lungs of mice with endotoxic shock.

### Administration of rPGRN protected WT and *Grn* ^−/−^ mice against endotoxic shock

**Figure 7:**
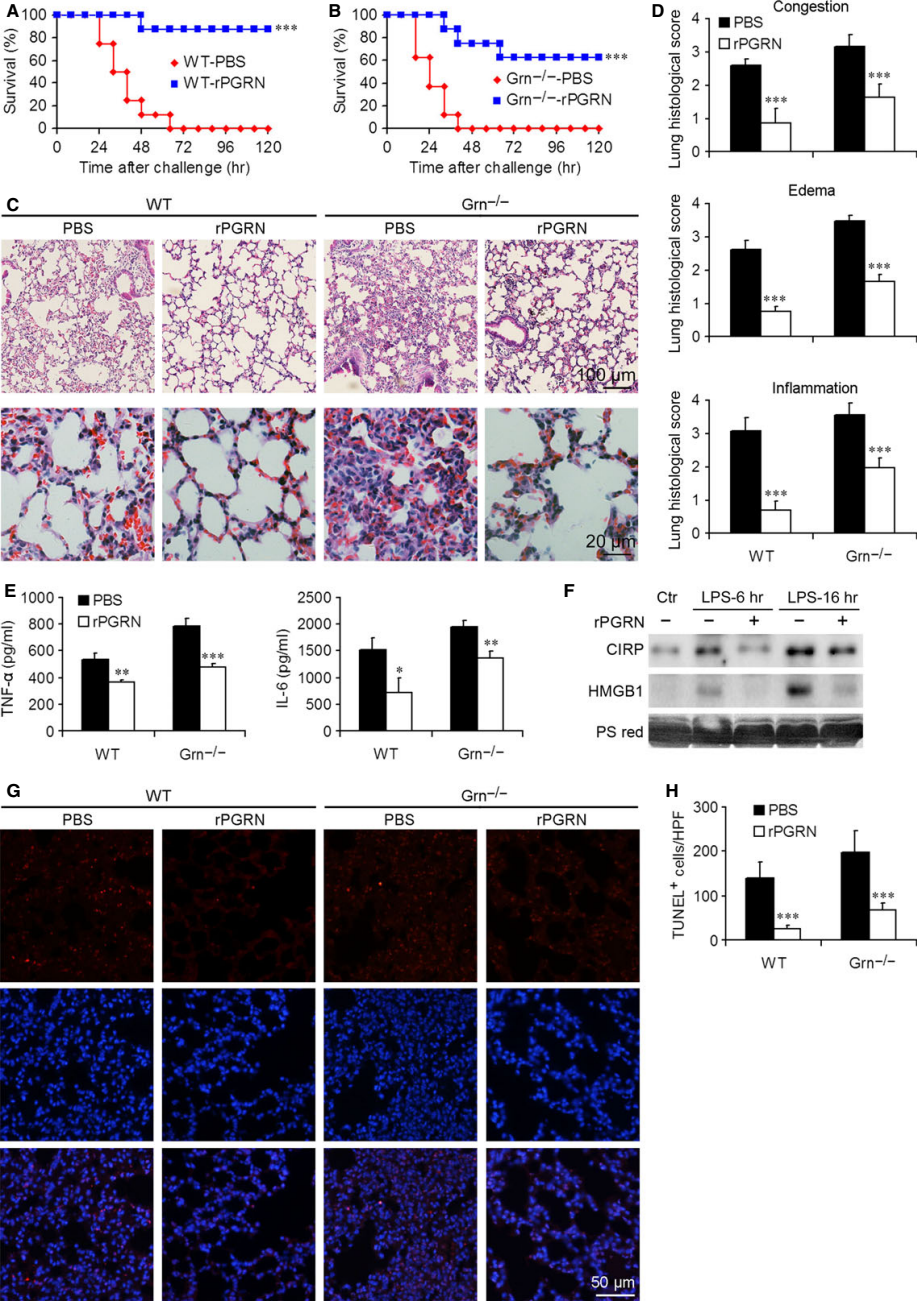
Pre‐treatment with rPGRN protected against endotoxic shock in WT and *Grn* ^−/−^ mice after LPS injection. Survival of (**A**) WT mice (*n* = 8 mice/group) and (**B**) *Grn* ^−/−^ mice (*n* = 8 mice/group) with and without rPGRN pre‐treatment. (**C** and **D**) Pathological changes in WT and *Grn* ^−/−^ lungs with and without rPGRN pre‐treatment. (**E**) Production of pro‐inflammatory cytokines in serum of WT and *Grn* ^−/−^ mice with rPGRN pre‐treatment. (**F**) Western blot analysis of CIRP and HMGB1 in the serum from WT mice with and without rPGRN pre‐treatment. PS red, Ponceau S red staining. (**G** and **H**) Apoptosis of lung cells in WT and *Grn* ^−/−^ endotoxic mice with and without rPGRN pre‐treatment. Data are mean ± S.D. *P < 0.05; **P < 0.01; ***P < 0.001 compared with WT or *Grn* ^−/−^ mice injected with LPS.

Because PGRN deficiency resulted in enhanced mortality and severe lung injury in mice with endotoxic shock, we further investigated the protective effect of PGRN in the endotoxic shock mouse model. Wild‐type and *Grn* ^−/−^ mice received rPGRN or PBS by intraperitoneal injection 2 hrs before the induction of endotoxic shock by LPS. Lipopolysaccharide at 35 mg/kg caused 100% mortality of WT and *Grn* ^−/−^ mice, whereas pre‐treatment with rPGRN at 10 mg/kg before LPS injection significantly improved the survival of both genotypes (Fig. 7A and B). Pre‐treatment with rPGRN markedly protected WT and *Grn* ^−/−^ mice against lung injury, as demonstrated by ameliorated lung damage at 16 hrs after LPS injection (Fig. 7C and D). Serum was obtained at 6 and 16 hrs after LPS injection for determining inflammatory mediator levels. Recombinant PGRN pre‐treatment significantly reduced the serum levels of TNF‐α and IL‐6 for both genotypes as compared with PBS treatment (Fig. 7E). Furthermore, rPGRN pre‐treatment also reduced the serum levels of CIRP and HMGB1 in WT mice at 6 and 16 hrs after LPS injection (Fig. 7F). In parallel with improved survival and reduced inflammation, rPGRN pre‐treatment attenuated lung cell apoptosis in WT or *Grn* ^−/−^ lungs after LPS injection at 16 hrs (Fig. 7G and H). Collectively, these results imply that PGRN was essential for conferring systemic and lung protection in endotoxic shock.

### PGRN reduced LPS‐induced inflammatory cytokine and chemokines production *in vitro*

**Figure 8:**
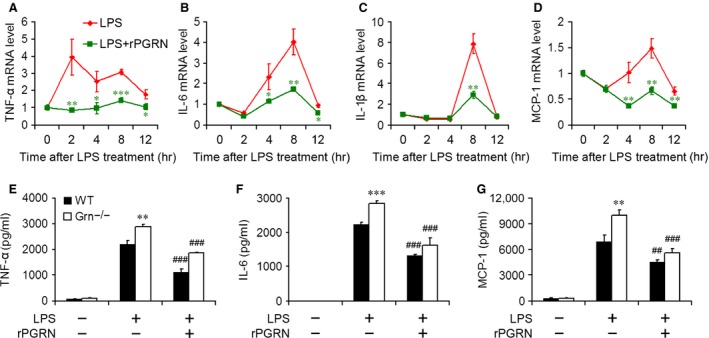
PGRN reduced LPS‐induced inflammatory reaction in A549 cells and BMDMs. Real‐time RT‐PCR analysis of relative TNF‐α (**A**), IL‐6 (**B**), IL‐1β (**C**) and MCP‐1 (**D**) mRNA level in A549 cells treated with 100 ng/ml LPS in the absence or presence of 500 ng/ml rPGRN. Data are mean ± S.D. *P < 0.05; **P < 0.01; ***P < 0.001 compared with cells treated with LPS alone. ELISA of level of TNF‐α (**E**), IL‐6 (**F**) and MCP‐1 (**G**) in medium of WT and *Grn* ^−/−^ BMDMs treated with 100 ng/ml LPS in the absence or presence of 500 ng/ml rPGRN. Data are mean ± S.D. **P < 0.01; ***P < 0.001 compared with WT BMDMs treated with LPS alone. ##*P* < 0.01; ###*P* < 0.001 compared with BMDMs treated with LPS alone.

The protective effect of PGRN on LPS‐induced inflammation was also studied *in vitro*. The real‐time RT‐PCR showed that LPS stimulated the mRNA levels of inflammatory cytokines and chemokines, including TNF‐α, IL‐6, IL‐1β and MCP‐1, in adenocarcinomic human alveolar basal epithelial A549 cells (Fig. 8A–D). Meanwhile, rPGRN treatment reduced LPS‐induced overexpression of inflammatory cytokines and chemokines in A549 cells (Fig. 8A–D). Bone marrow–derived macrophages isolated from WT and *Grn* ^−/−^ mice were treated with LPS in the absence or presence of rPGRN for 24 hrs, and culture medium was collected for ELISA. More TNF‐α, IL‐6 and MCP‐1 were released from *Grn* ^−/−^ macrophages compared with those from WT macrophages, rPGRN treatment reduced LPS‐induced production of pro‐inflammatory cytokines and chemokines in WT and *Grn* ^−/−^ macrophages (Fig. 8E–G).

## Discussion

Mounting evidence demonstrates that PGRN plays important roles in the inflammatory response 22. However, the biological function of PGRN in endotoxic shock remains unclear. We aimed to examine the potential role of PGRN in the pathogenesis of endotoxic shock using PGRN‐deficient mice. Progranulin‐deficient mice showed severe endotoxic shock when challenged with LPS. Moreover, rPGRN pre‐treatment had protective effects against LPS‐induced endotoxic shock in mice.

The elevated expression of PGRN has been reported in inflammation‐related diseases or models, such as blood and adipose tissues in a mouse obesity model 14, cartilage of patients with arthritis as compared with normal controls 23, colons from inflammatory bowel‐diseased patients and a mouse colitis model 24 and gastric epithelial cells after infection with *Helicobacter pylori* 25, 26. Here, we evaluated the serum and lung levels of PGRN in mice with endotoxic shock. Serum protein levels of PGRN and mRNA and protein levels in lung homogenates were increased at 6 and 24 hrs after LPS challenge in WT mice. However, intracellular PGRN level in lungs was increased at 6 hrs, followed by a decrease at 24 hrs after LPS administration, which suggests enhanced PGRN secretion from lung cells in the early stage of LPS‐induced shock. Progranulin might be an important growth factor in the endotoxin‐induced lung system and the local inflammatory response and damage. This result is consistent with findings that PGRN levels in bronchoalveolar lavage fluid were increased at day 1 and decreased at day 3 after LPS challenge in mice with LPS‐induced acute lung injury 22.

Gram‐negative bacteria and/or their products can active the immune system and result in a cascade of events leading to endotoxic shock 27. In our experiments, PGRN deficiency exacerbated the severity of endotoxic shock induced by LPS administration. *Grn* ^−/−^ mice were significantly more susceptible to endotoxic shock‐associated mortality, which indicates a critical role of PGRN in protecting against LPS‐induced shock. In addition, rPGRN prominently alleviated the symptoms of LPS‐induced shock and lung injury, which was confirmed by clinical and histological parameters in WT and *Grn* ^−/−^ mice.

The innate immune response constitutes the first line of defence against invading microbial pathogens and relies on phagocytes, such as granulocytes and macrophages. Neutrophils are critical for host defence and are recruited to the foci of bacterial infection regularly and promptly, thereby generating large quantities of reactive oxygen species (ROS) 28 and releasing granular contents to kill pathogens 29. Macrophages play major roles in the response to invading pathogens and release many pro‐inflammatory mediators, including nitric oxide, prostaglandin E2 and cytokines such as TNF‐α, IL‐1β and IL‐6 30. Thus, we explored the accumulation of neutrophils and macrophages in the endotoxic mouse lung and production of pro‐inflammatory mediators in WT and *Grn* ^−/−^ mice exposed to LPS. Progranulin deficiency enhanced the levels of pro‐inflammatory mediators, including TNF‐α and IL‐6, in serum and lung tissues of endotoxic mice. Cold‐inducible RNA‐binding protein, a member of cold shock protein family, has been recently identified as an endogenous inflammatory mediator that promotes inflammatory response in animal models of haemorrhagic shock, sepsis and liver ischaemia/reperfusion injury 31, 32. Recombinant CIRP stimulates the release of TNF‐α and HMGB1 from macrophages 31. High mobility group box protein 1 has been identified as a late mediator of endotoxin mortality in mice and regarded as a member alarmin danger signals in triggering immune responses 33, 34. Here, we assessed the impact of PGRN deficiency on circulating levels of CIRP and HMGB1 during endotoxic shock. Serum protein levels of CIRP and HMGB1 were increased after LPS challenge in WT and *Grn* ^−/−^ mice. As compared with endotoxic WT mice, endotoxic *Grn* ^−/−^ mice displayed higher elevation of serum CIRP and HMGB1. Conversely, rPGRN pre‐treatment significantly reduced the serum levels of TNF‐α and IL‐6 in endotoxic WT and *Grn* ^−/−^ mice and the serum levels of CIRP and HMGB1 in endotoxic WT mice. Consistently, the accumulation of neutrophils and macrophages was further increased in lungs of endotoxic *Grn* ^−/−^ mice, so loss of PGRN signalling exacerbated systemic and local inflammation.

The protective role of PGRN in LPS‐induced inflammation was also confirmed *in vitro* as evidenced by reduced production of pro‐inflammatory cytokines and chemokines in LPS‐treated human alveolar basal epithelial cells and WT and *Grn* ^−/−^ BMDMs in the presence of rPGRN, comparing with cells treated with LPS alone. In addition, pre‐treatment with rPGRN reduced LPS injection induced overexpression of pro‐inflammatory mediators in kidney and histological lesions in kidney and liver (unpublished data), indicating a generally protective role of PGRN against multi‐organ failure during endotoxic shock.

Mediators of the endotoxic response, such as TNF‐α, intracellular calcium accumulation, nitric oxide and increased levels of ROS, ultimately cause cell death by necrosis and apoptosis 35. In this study, we found that PGRN deficiency led to severe apoptosis in lungs of endotoxic mice, and levels of apoptosis‐associated Bax, cleaved caspase 3, cytochrome c and cleaved PARP1 were elevated in lungs of endotoxic *Grn* ^−/−^ mice. Progranulin is a well‐accepted survival factor for normal and cancer cells *in vitro* 36. As a stress‐response factor, PGRN protects fibroblasts against hypoxia and acidosis‐induced cell death 37. Our previous study indicated that PGRN inhibited ischaemia/reperfusion‐induced apoptotic cell death of renal cells *in vitro* and *in vivo* 18. In this study, we found that rPGRN pre‐treatment protected against cell death in lung tissues of WT and *Grn* ^−/−^ mice with endotoxic shock.

Although the detailed mechanisms underlying PGRN‐mediated protective effects in endotoxic shock are not clear, several findings suggest that PGRN regulates LPS‐induced production of inflammatory mediators and clearance of intracellular bacteria 17 and that PGRN inhibits inflammation induced by TNF‐α 38. Recent studies have shown that PGRN level is associated with the receptors TNFR1, TNFR2 and DR3, members of the TNF receptor superfamily 10, 12, 39, 40, by which PGRN suppresses inflammation in various kinds of conditions 12, 13, 22, 38, 41, 42, 43, 44. In addition to binding to TNFRs, PGRN binds to TLR9 and mediates the recruitment of CpG oligodeoxynucleotides in macrophages, which suggests a potential role of PGRN in innate immunity against microorganisms 45.

In conclusion, our findings demonstrate that PGRN is overexpressed in the early stage of endotoxic shock in mice, which may represent a self‐protective effect on endotoxic shock, because *Grn* ^−/−^ mice showed severe systemic and local inflammatory responses and tissue injury as compared with WT mice after LPS injection. The protective function of PGRN also has important therapeutic implications in reducing morbidity and mortality from endotoxic shock.

## Conflicts of interest

The authors confirm that there are no conflicts of interest.
